# Assessment of Systemic Toxicity, Genotoxicity, and Early Phase Hepatocarcinogenicity of Iron (III)-Tannic Acid Nanoparticles in Rats

**DOI:** 10.3390/nano12071040

**Published:** 2022-03-22

**Authors:** Chi Be Hlaing, Arpamas Chariyakornkul, Chalermchai Pilapong, Charatda Punvittayagul, Somdet Srichairatanakool, Rawiwan Wongpoomchai

**Affiliations:** 1 Department of Biochemistry, Faculty of Medicine, Chiang Mai University, Chiang Mai 50200, Thailand; hlaingchibe26o@gmail.com (C.B.H.); arpamas.ch@cmu.ac.th (A.C.); somdet.s@cmu.ac.th (S.S.); 2 Center of Excellence for Molecular Imaging (CEMI), Department of Radiologic Technology, Faculty of Associated Medical Sciences, Chiang Mai University, Chiang Mai 50200, Thailand; chalermchai.pilapong@cmu.ac.th; 3 Research Affairs, Faculty of Veterinary Medicine, Chiang Mai University, Chiang Mai 50100, Thailand; charatda.pun@cmu.ac.th

**Keywords:** acute toxicity, repeated dose toxicity, carcinogenicity, genotoxicity, nanoparticle

## Abstract

Iron-tannic acid nanoparticles (Fe-TA NPs) presented MRI contrast enhancement in both liver cancer cells and preneoplastic rat livers, while also exhibiting an anti-proliferative effect via enhanced autophagic death of liver cancer cells. Hence, a toxicity assessment of Fe-TA NPs was carried out in the present study. Acute and systemic toxicity of intraperitoneal Fe-TA NPs administration was investigated via a single dose of 55 mg/kg body weight (bw). Doses were then repeated 10 times within a range of 0.22 to 5.5 mg/kg bw every 3 days in rats. Furthermore, clastogenicity was assessed by rat liver micronucleus assay. Carcinogenicity was evaluated by medium-term carcinogenicity assay using glutathione *S*-transferase placental form positive foci as a preneoplastic marker, while three doses ranging from 0.55 to 17.5 mg/kg bw were administered 10 times weekly via intraperitoneum. Our study found that the LD_50_ value of Fe-TA NPs was greater than 55 mg/kg bw. Repeated dose administration of Fe-TA NPs over a period of 28 days and 10 weeks revealed no obvious signs of systemic toxicity, clastogenicity, and hepatocarcinogenicity. Furthermore, Fe-TA NPs did not alter liver function or serum iron status, however, increased liver iron content at certain dose in rats. Notably, antioxidant response was observed when a dose of 17.5 mg/kg bw was given to rats. Accordingly, our study found no signs of toxicity, genotoxicity, and early phase hepatocarcinogenicity of Fe-TA NPs in rats.

## 1. Introduction

The global cancer burden has rose to 19.3 million new cases and 9.9 million deaths in 2020. Primary liver cancer is the sixth most common cause of cancer and the third leading cause of cancer mortality worldwide [1]. About 80% of primary liver cancer cases involve hepatocellular carcinoma (HCC). The high rate of mortality observed in HCC patients can be attributed to late diagnosis, drug resistance, disease recurrence, and metastasis leading to poor clinical outcomes [2].

Imaging is one of the most important aspects of diagnosis, therapy, and follow-up for HCC patients [3]. Magnetic resonance imaging (MRI), or multiphase contrast-enhanced computed tomography (CT), is recommended for initial diagnostic evaluation of clinically suspicious HCC. The therapeutic options depend upon the stage of the disease and what curative and noncurative interventions are available. Curative forms of therapy include surgical resection, orthotopic liver transplant, and ablative techniques such as thermal ablation. Noncurative forms of therapy include transarterial chemoembolization (TACE), transarterial radioembolization (TARE), stereotactic body radiation therapy (SBRT), and systemic chemotherapy [4]. However, early diagnosis and effective treatment have remained challenging with regard to HCC management.

The use of nanomaterials in cancer management is one of the most promising and advanced approaches. Many studies have reported that nanoparticles (NPs) can play a role in the diagnosis and treatment of cancer [5]. Alshehri S et al. have recently reviewed current studies on theranostic applications of nanoparticles and reported that these studies reported favourable outcomes in both in vitro and in vivo cancer models [6]. In addition, many research studies are now focused on the application of nanodrug delivery systems for diagnostic and therapeutic agents in liver cancer patients in order to improve clinical efficacy [7]. Among the various types of NPs, metal-polyphenol nanoparticles exhibit a potential role in cancer theranostics. Polyphenols, such as tannic acid (TA), gallic acid, and epigallocatechin gallate, can assemble with certain metal ions, such as Fe^3+^, Mn^2+^, and Cu^2+^, and then be loaded with chemotherapeutic agents or exist in their pure form. These nanoparticles have been studied in tumor imaging studies that involved magnetic resonance imaging (MRI), CT imaging, photothermal imaging, photoacoustic imaging, as well as tumor therapy photothermal therapy, photodynamic therapy, and chemotherapy [8]. In this context, metals and polyphenols can display their biological effects via individual or synergistic actions for both diagnostic and therapeutic purposes in cancer treatments [9]. Self-assembled ferric and TA nanoparticles (Fe-TA NPs) can provide MRI signals in hepatocellular carcinoma cells [10] and rat liver preneoplasia models [11]. Furthermore, Fe-TA NPs also showed an antiproliferative effect via enhanced autophagic cell death in hepatocellular carcinoma cells [10]. This finding highlights the potential of Fe-TA NPs as a nanotheranostic agent for early diagnosis and treatment of HCC.

However, the potential toxicity of nanoparticles is of concern as there is limited available knowledge on the toxicity of self-assembled metal-polyphenol theranostic nanoparticles in vivo [6]. Furthermore, a dose–response relationship is an integral factor that is involved in evaluating the toxicity of a chemical substance. Some physical and chemical agents such as low-dose ionizing radiation and non-genotoxic carcinogens, including phenobarbital and α-benzene hexachloride, as well as certain phytochemicals such as resveratrol, quercetin, and sulforaphane, have exhibited a hormetic dose response [12,13,14]. To explore the further diagnostic and therapeutic potential of Fe-TA NPs, the present study assessed the toxicity of Fe-TA NPs through single and repeated dose systemic toxicity tests in rats, in vivo genotoxicity tests, and medium-term carcinogenicity test in rats. 

## 2. Materials and Methods

### 2.1. Chemicals

Diethylnitrosamine (DEN), 3,3′-diaminobenzidine tetrahydrochloride hydrate (DAB), 5,6-diphenyl-1,2,4-triazine-p, p′-disulfonic acid disodium salt hydrate (ferrozine), and butylated hydroxytoluene (BHT) were purchased from Sigma-Aldrich (St. Louis, MO, USA). Collagenase type IV and 4′,6-diamidino-2-phenylindole (DAPI) were obtained from Invitrogen (Waltham, MA, USA). Assay kits for serum iron (RX SERIES SI 382) and serum TIBC (RX SERIES TI 3858) were purchased from Randox Laboratories Ltd. (Antrim, Northern Ireland, UK). Ferritin Rat ELISA Kit (ab157732) was purchased from Abcam (Cambridge, UK). Anti-rat GST-placental form was obtained from MBL (Nagoya, Japan) and mouse monoclonal purified anti-rat PCNA antibody was obtained from BioLegend (San Diego, CA, USA). ApopTag Peroxidase In Situ Apoptosis Detection Kit (S7100) for TUNEL assay was purchased from Merck KGaA (Darmstadt, Germany). EnvisionTM G/2 Doublestain System, Rabbit/Mouse (DAB+/Permanent Red) was obtained from Dako (Glostrup, Denmark). Vectastain ABC kit was obtained from Vector Laboratories, Inc. (Burlingame, CA, USA). β-NADPH was purchased from Oriental Yeast (Tokyo, Japan) and glucose-6-phosphate dehydrogenase was purchased from Worthington Biochemical Corporation (Lakewood, NJ, USA). Reduced and oxidized glutathione, glutathione reductase, 5,5′-dithiobis (2-nitrobenzoic acid (DTNB), glucose-6-phosphate, and hemin were obtained from Sigma-Aldrich, (St. Louis, MO, USA). All other chemicals were analytical grade.

### 2.2. Iron (III)—Tannic Acid Nanoparticles 

Fe-TA NPs were obtained from Dr. Chalermchai Pilapong, Center of Excellence for Molecular Imaging, Department of Radiologic Technology, Faculty of Associated Medical Sciences, Chiang Mai University, Chiang Mai, Thailand. Fe-TA NPs are known to exist in stable tris-coordinated complexes in PBS buffer (pH 7.4). Fe-TA NPs are spherical in shape with a hydrodynamic diameter of 3.14 ± 1.0 nm. Fe-TA NPs present a zeta potential of −23 ± 2.1 mV and exhibit good colloidal stability, water solubility, and high stability against both transchelation and transmetallation. The quantity of Fe-TA NPs was expressed as an equivalent concentration of iron spectrophotometrically [10]. 

### 2.3. Animal Studies

Wistar rats (5 to 7-week-olds) were obtained from Nomura Siam International Co., Ltd., Bangkok, Thailand. Rats were housed in stainless steel cages and acclimated for a week before beginning investigations. Housing conditions included a 12:12-h light/dark cycle at temperatures of 23 ± 2 °C and the level of humidity maintained within a range of 50 to 60%. Both a pellet diet and water were provided ad libitum. The animal protocols used in this study followed the relevant international and national guidelines for animal use and were approved of by The Animal Ethics Committee of the Faculty of Medicine, Chiang Mai University, Thailand (numbers: 39/2560 and 06/2563). 

### 2.4. Single Dose Toxicity Test

Eight-week-old male Wistar rats were randomly divided into 2 groups, control and Fe-TA NPs treated groups, with 5 animals in each group. Fe-TA NPs were injected intraperitoneally at a dose of 55 mg/kg body weight a maximum dose for its solubility. Animals were observed for a total of 14 days for any sign of toxicity or mortality. Body weights of the rats were recorded twice a week. Animals were sacrificed under isoflurane inhalation on day 14 of the experiment. The livers, kidneys, and spleens of the animals were collected and weighed. Hematological parameters, including red blood cell count, leucocyte count, and platelet count, were determined with the use of an automatic analyzer at the Veterinary Diagnostic Centre Co., Ltd. (Chiang Mai, Thailand).

### 2.5. Repeated Dose Toxicity Test

Six-week-old male and female Wistar rats were randomly allocated into 6 groups with 10 animals in each group (5 rats of each gender). Group 1 served as a vehicle control group. Groups 2–4 were administered with Fe-TA NPs at doses of 0.22, 1.1, and 5.5 mg/kg bw, respectively. The remaining two groups were satellite groups, and were comprised of a control and a group that received 5.5 mg/kg bw of Fe-TA NPs. Fe-TA NPs were injected intraperitoneally every three days for a total of 10 times. Test doses of Fe-TA NPs were selected based on the single dose toxicity test and aimed to achieve a cumulative dose of 55 mg/kg bw. The dosage interval was based on an established liver clearance time of Fe-TA NPs [15]. Rats were monitored by measuring body weight every three days and food and water intake twice per week. After 24 h of the last injection and overnight fasting, rats were sacrificed under isoflurane inhalation. For the satellite groups, this duration was extended for a period of 14 days after the last injection, while body weight, food, and water intake were monitored according to the method that had been previously explained. Rats were observed for any delayed or persistent effect of, or recovery from, toxic effects during that period. Whole blood was collected for hematological and biochemical investigations in the Veterinary Diagnostic Laboratory, Faculty of Veterinary Medicine, Chiang Mai University and the Veterinary Diagnostic Centre Co. Ltd., Chiang Mai, Thailand. Urine samples were taken for urinalysis using a dipstick test (Multistix 10 SG, Siemens, Melbourne, Australia). Vital internal organs were removed for gross necropsy and organ weight measurement before being fixed in buffered 10% formalin for histopathological assessment. The remaining portion of the liver was kept in −20 °C until further analysis.

### 2.6. Clastogenicity Test

**Figure 1:**
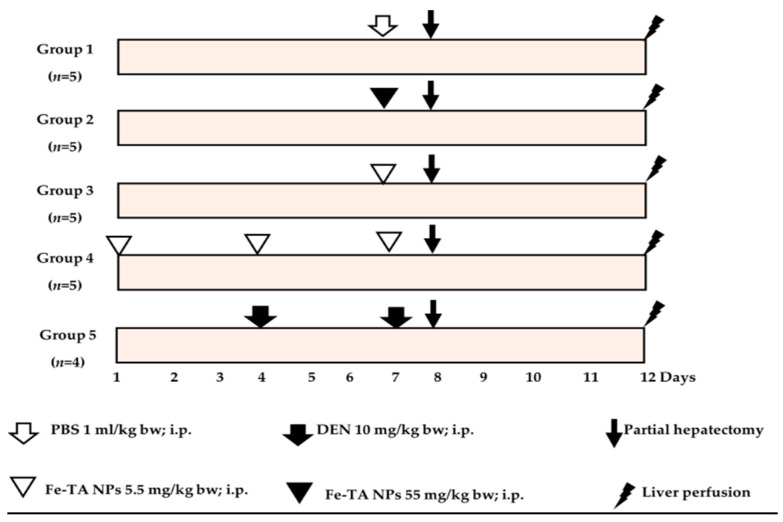
Experimental protocol for clastogenicity test of Fe-TA NPs in rats.

To determine the clastogenic activity of intraperitoneal Fe-TA NP administration, rat liver micronucleus assay was used in this study. Male Wistar rats (6-week-old, 180–190 g) were divided into five groups as is shown in Figure 1. Group 1 was established as a negative control receiving phosphate buffered saline, while groups 2 and 3 received a single dose of Fe-TA NPs at 55 and 5.5 mg/kg bw, respectively. Group 4 rats were injected with repeated doses of 5.5 mg/kg bw of Fe-TA NPs on days 1, 4 and 7 of the experiment. Group 5 served as a positive control receiving 10 mg/kg bw of diethylnitrosamine (DEN) on days 4 and 7 of the experiment. Twenty-four hours after the last injection, all rats were lightly anesthetized with isoflurane inhalation and underwent 2/3 partial hepatectomy. After the experiment was conducted for 4 days, rats were euthanized by overdose thiopental and their hepatocytes were isolated using the 2-step collagenase perfusion technique. Liver cells were stained with 4′,6-diamidino-2-phenylindole (DAPI) and analyzed under a fluorescent microscope. Micronucleated and mitotic hepatocytes were observed as 2000 hepatocytes/rat. The criteria for micronucleated hepatocyte scoring had been previously established by Chariyakornkul et al., 2019 [16].

### 2.7. Medium-Term Liver Carcinogenicity Test

Six-week-old male Wistar rats were divided into 5 groups. Group 1 was a negative control receiving phosphate buffered saline and groups 2–4 (*n* = 4–6) served as treated groups that were intraperitoneally injected with Fe-TA NPs at doses of 0.55, 5.5 and 17.5 mg/kg bw once a week on 10 occasions, while group 5 (*n* = 3) was considered a positive control that received weekly injections of diethylnitrosamine at 100 mg/kg body weight on three occasions. One week before injections were given, all animals underwent two-third partial hepatectomies to induce regenerative cell proliferation. Twenty-four hours after the last injection was administered, all rats were sacrificed under isoflurane inhalation and blood sampling was collected. Serum alanine aminotransferase (ALT) was collected after the clotted blood was centrifuged and analyzed using an automatic analyzer at the Veterinary Diagnostic Centre Co., Ltd., Chiang Mai, Thailand. Subsequently, livers were perfused with 0.15 M KCl to remove blood samples. Livers, spleens, and kidneys of the rats were then collected and weighed. Liver portions were fixed in 10% buffered formalin and then embedded in paraffin for histopathological assessment of the formation of GST-P positive foci as a preneoplastic lesion in the livers, proliferating cell nuclear antigen (PCNA)-positive cells as cell proliferation markers and apoptotic cells by terminal deoxynucleotidyl transferase (TdT) dUTP nick end labelling (TUNEL) assay. These procedures were performed according to the method previously described [17].

### 2.8. Measurement of Serum and Liver Iron Status Parameters

The levels of serum iron and total iron-binding capacity were measured colorimetrically using commercial assay kits and a fully automatic biochemical analyzer (RX Daytona, Antrim, Ireland) according to the manufacturer’s instructions. The percentage of transferrin saturation was calculated as a ratio of serum iron to TIBC.

Liver iron concentration was determined using a modified ferrozine assay as has been described elsewhere [18]. After dried liver tissues were cold homogenized in 50 mM phosphate buffer at a pH of 2.0 containing 0.005% methanolic BHT, they were further centrifuged and the supernatant was collected. The iron standards were prepared from a serial dilution of ferrous ammonium sulfate (0–800 μM). Standards and the supernatant were incubated with a chromogenic solution containing 0.508 mM ferrozine, 1.5 M sodium acetate, and 1.5% (*v*/*v*) thioglycolic acid at room temperature for 30 min. Absorbance was measured spectrophotometrically at 562 nm. The concentration of liver iron was calculated from the standard curve and expressed as ng of iron per mg of dry liver weight.

Liver ferritin values were determined using a Ferritin Rat ELISA Kit. Liver tissue samples were homogenized with ice cold NaCl and then centrifuged at 15,000 rpm at 4 °C for 20 min. The supernatant was collected and diluted with diluent buffer. A standard curve was prepared with a serial dilution of rat ferritin calibrator (0–100 ng/mL), and then ferritin levels were measured according to the manufacturer’s instructions. Liver ferritin concentration values were calculated using a calibration curve that was normalized by protein concentrations determined by Lowry’s method [19] and then expressed as a value of ng/mg liver protein.

### 2.9. Determination of Antioxidant Parameters in Rat Livers

Total glutathione (GSH) and glutathione disulfide (GSSG) levels were determined using the enzymatic recycling method based on DTNB with some modifications [20]. Liver homogenate in 0.01 M phosphate buffer was treated with 5% 5-sulfosalicylic acid. After cold centrifugation, supernatants were reacted with 4-vinylpyridine to protect GSH auto-oxidation for GSSG assay and then used directly for total GSH determination. The standards or samples were mixed with a reaction mixture containing glutathione reductase, DTNB, and β-NADPH. The degree of absorbance was measured kinetically at 405 nm for 5 min with a minute interval. The concentration was calculated with the use of a regression curve generated from the standards of GSH and GSSG and expressed as a value of nmol/mg protein. Reduced glutathione levels were calculated by subtracting values of GSSG from total GSH.

For the determination of antioxidant enzyme activity, the liver tissue was homogenized on ice with homogenizing buffer containing 1.15% (*w*/*v*) KCl and 0.25 mM PMSF. After medium-speed centrifugation, the supernatant was subsequently centrifuged at 30,000 rpm for 60 min at 4 °C in order to obtain cytosolic and microsomal fractions. The protein content of both fractions was then determined by applying the Lowry method using bovine serum albumin (BSA) as a standard [19].

Catalase (CAT) activity was determined using the method described by Aebi, 1984 [21]. In this assay, 30 mM H_2_O_2_ was used as a substrate and catalase activity was detected by a decrease in absorbance of H_2_O_2_ at 240 nm. CAT activity was calculated using molar extinction coefficient for H_2_O_2_ at 240 nm and expressed as µmol of H_2_O_2_ per minute per mg protein.

Glutathione reductase (GR) activity was determined according to the method previously described by Carlberg and Mannervik, 1985 [22]. Assay samples were reacted with a reaction mixture that contained phosphate buffer (pH 7.0) prepared from 0.1 M KH_2_PO_4_, 1 mM MgCl_2_.6H_2_O, 25 mM GSSG, and 0.1 mM NADPH. The oxidation of NADPH during the reaction was estimated spectrophotometrically at 340 nm for a single minute. GR activity was expressed in terms of µmol of NADPH consumed per minute per mg protein using the molar extinction coefficient for NADPH at 340 nm.

Glutathione peroxidase (GPx) activity was determined with slight modifications according to the method described by Nagalakshmi and Prasad (2001) [23]. Assay samples were added to the reaction mixture containing 0.1 M Tris-EDTA buffer (pH 8), 0.1 M GSH, 2 mM β-NADPH, 7 mM t-BHP, and 10 U GR. β-NADPH was oxidized during the reaction and was measured by estimating a decrease in the absorbance at 340 nm. GPx activity was calculated using the molar extinction coefficient for NADPH at 340 nm and was expressed in terms of µmol of NADPH consumed per minute per mg protein.

Heme oxygenase (HO) activity assay was performed using a slightly modified method [24]. The liver microsome was incubated with 0.1 M potassium phosphate buffer containing 2 mM MgCl_2_ at a pH of 7.4 with 8 mM glucose-6-phosphate, 4 U/mL glucose-6-phosphate dehydrogenase, 6.4 mM NADPH, 400 µM hemin substrate, and 10 mg/mL of liver cytosol as a source of biliverdin reductase at 37 °C in the dark. The reaction was stopped using chloroform. The activity of HO was measured at wavelengths of 460 and 530 nm and expressed as nmol/min/mg protein using a molar extinction coefficient of 40.

### 2.10. Statistical Analysis

Data are presented as mean ± standard deviation values (S.D) for each variable. Statistical differences among three or more groups were assessed by one-way analysis of variance (ANOVA) followed by Bonferroni as a post-hoc test for toxicity tests and least significant differences for the relevant biochemical parameters. Statistical differences between the two groups were analyzed by Student’s *t*-test. Data were considered statistically significant when a *p*-value of less than 0.05 was observed.

## 3. Results

### 3.1. Single and Repeated Dose Toxicity Levels of Iron (III)–Tannic Acid Nanoparticles in Rats

Single dose toxicity test aims to estimate the median lethal dose (LD_50_) of Fe-TA NPs in rats. During the 14-day observation period, a single intraperitoneal injection of 55 mg/kg bw of Fe-TA NPs was not found to have caused any toxic symptoms or mortality. The gross necropsy indicated a non-disorder in organ morphology. Body weight, absolute and relative organ weights, and hematological parameters, including red blood cell, leucocyte, and platelet counts, were not found to be significantly different when compared to the control group (data not shown). It was indicated that the LD_50_ value of intraperitoneal Fe-TA NPs administration was more than 55 mg/kg body weight in male Wistar rats.

The repeated dose test aims to assess the most affected target organs and dosages of the test substance after a certain period of continuous exposure. The intraperitoneal treatment of Fe-TA NPs ranged from 0.22 to 5.5 mg/kg bw every 3 days for 10 times. This indicated no incidences of morbidity and mortality in both genders of rats. No significant changes in food and water intake and body weight were observed in both sexes of the experimental rats when compared to the control animals (Table 1). The absolute and relative organ weights of ovaries in 5.5 mg/kg bw dose groups and those of the seminal vesicles in 1.1 mg/kg bw dose groups were significantly increased when compared to those of the control group. However, no histopathological changes were observed. Apart from these findings, there were no significant changes in organ weight of Fe-TA NPs treated animals when compared to the control animals of both sexes (Table 1). In the satellite group of female rats, the brain weights of the rats in the Fe-TA NPs treated group were significantly reduced when compared to the control group (Table 1). No other significant changes were observed in terms of gross necropsy and organ weights of the members of both sexes of the satellite group (Table 1).

**Figure 2:**
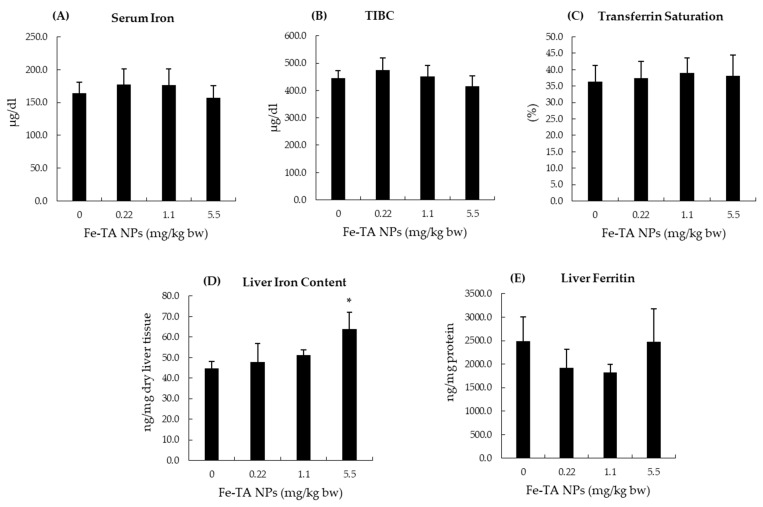
Effect of 28-day experiment of Fe-TA NPs on serum and liver iron parameters in male rats. (**A**): Serum iron, (**B**): TIBC (Total iron binding capacity), (**C**): Transferrin saturation, (**D**): Liver iron content, and (**E**) Liver ferritin. Data are expressed as Mean ± SD. * *p* < 0.05, significantly different when compared with a control group.

The systemic toxic effects of Fe-TA NPs in rats were further studied. The hematological profile of female rats did not reveal any significant changes when compared to the control rats (Table 2). In male rats, the neutrophil count was significantly increased in groups receiving 1.1 and 5.5 mg/kg bw doses, while monocyte counts were significantly reduced in groups receiving 0.22 and 1.1 mg/kg bw doses when compared to the control group (Table 2). However, these values were found to be within the reference range. Although the biochemical profiles of female rats revealed significant reductions in BUN levels in both the medium- and high-dose treated groups, these levels were also determined to be within the reference range (Table 3). In male rats, serum potassium levels were significantly increased in the group receiving 5.5 mg/kg bw doses (Table 3). Moreover, significant changes in levels of creatinine, sodium, and TCO_2_ in the satellite group of Fe-TA NPs treated male rats were also found to be within the reference range (Table 3). No significant differences were observed in liver function tests in terms of lipid profiles and blood glucose levels in rats of both sexes (Table 3). Urinalysis revealed no abnormalities apart from hematuria in some samples obtained from female rats. However, various levels of proteinuria in male rats were detected in most of the urine samples, while hematuria and ketonuria were detected in some of the other samples. Furthermore, the effects of Fe-TA NPs on serum and liver iron status were assessed in male rats. Serum iron, serum TIBC, and transferrin saturation values in the treated groups were not changed significantly when compared to the vehicle control groups. Fe-TA NPs treatment significantly increased liver iron content at 5.5 mg/kg bw dose; however, ferritin content between the treated groups and the vehicle control group were not determined to be different (Figure 2).

### 3.2. Clastogenic Effect of Iron-Tannic Molecular Nanoparticles in Rats

The clastogenic effect of Fe-TA NPs was assessed through rat liver micronucleus assay, which served as the primary test in a battery of genotoxicity tests. Micronuclei can be formed during cell division from chromosome fragmentation or as a consequence of a failure to be incorporated into the daughter nuclei [25]. A single or repeated treatment of Fe-TA NPs did not affect water and food intake (data not shown) or result in body weight gains (Table 4). No evidence of a significant increase was observed in the number of micronucleated hepatocytes, binucleated hepatocytes, and mitotic index values after either a single dose or repeated doses of 5.5 mg/kg bw of Fe-TA NPs. In addition, no statistical differences were observed in these parameters from a single treatment of Fe-TA NPs in terms of its LD_50_ value at a dose of 55 mg/kg bw in rats of both genders. On the other hand, an injection of DEN, a positive control, produced a significant increase in the number of micronucleated hepatocytes and the mitotic index value (7.30 + 2.19 and 2.56 + 0.28 per 1000 hepatocytes) when compared to the negative control. These findings indicate that Fe-TA NPs did not result in incidences of clastogenicity in rats.

### 3.3. Carcinogenicity of Iron (III)–Tannic Acid Nanoparticles in Rats

The medium-term carcinogenicity test is a reliable and practical tool to predict the carcinogenic potential of chemicals compared to the others. Immunohistochemically detected glutathione *S*-transferase placental form (GST-P) positive focus as the end-point marker can be used as early indicator of preneoplastic lesions in rat liver [26]. Although no abnormal clinical features were observed in the rats, one rat died after receiving 7 doses of 17.5 mg/kg bw of Fe-TA NPs. No significant changes in body weight, the weight of some vital organs, and serum ALT levels were observed (Table 5). With regard to the liver preneoplastic marker, diethylnitrosamine, a hepatocarcinogen, could induce the number and area of GST-P positive foci (14.22 ± 3.77 foci and 1.55 ± 0.56 mm^2^ per liver area (cm^2^), respectively), whereas Fe-TA NPs at various doses did not induce GST-P positive foci formation in rat livers (Table 5). Furthermore, the administration of Fe-TA NPs ranged from 0.55–17.5 mg/kg bw did not alter the number of PCNA-positive and apoptotic cells in the livers. Accordingly, it might be suggested that the administration of intraperitoneal Fe-TA NPs up to 17.5 mg/kg bw, once a week for 10 times, was not carcinogenic to the livers of rats.

### 3.4. Effect of Iron (III)–Tannic Acid Nanoparticles on Some Biochemical Parameters in Serum and Livers of Rats

**Figure 3:**
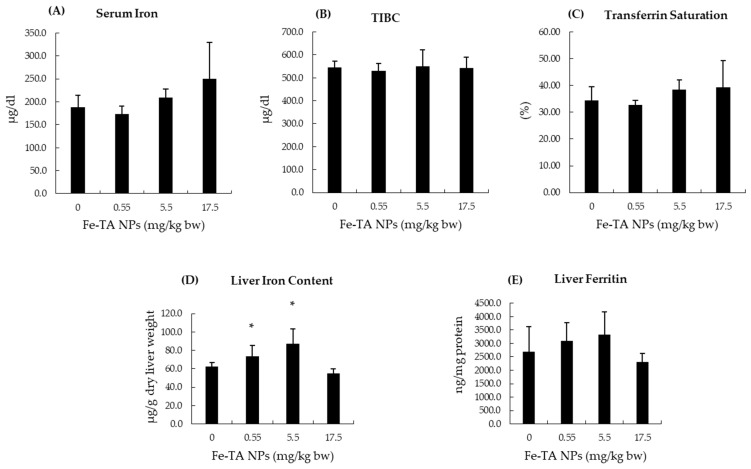
Effect of 10-week experiment of Fe-TA NPs on serum and liver iron parameters in rats. (**A**): Serum iron, (**B**): TIBC (Total iron binding capacity), (**C**): Transferrin saturation, (**D**): Liver iron content, and (**E**) Liver ferritin. Data are expressed as Mean ± SD. * *p* < 0.05, significantly different when compared with a control group.

**Figure 4:**
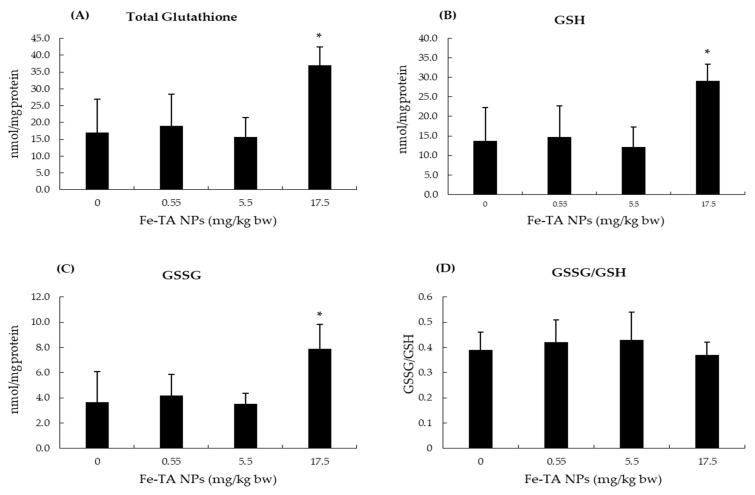
Effect of 10-week experiment of Fe-TA NPs on hepatic glutathione content in rats. (**A**): Total glutathione, (**B**): GSH (Reduced glutathione), (**C**): GSSG (Glutathione disulfide), and (**D**): GSSG/GSH (Glutathione disulfide to reduced glutathione ratio). Data are expressed as Mean ± SD. * *p* < 0.05, significantly different when compared with a control group.

**Figure 5:**
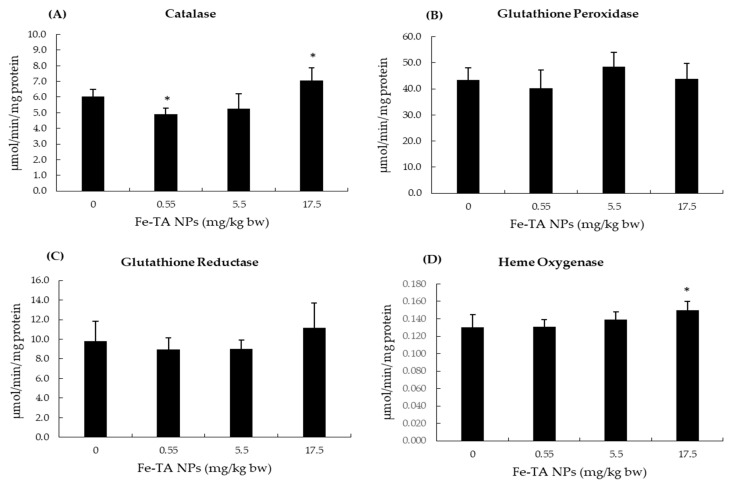
Effect of 10-week experiment of Fe-TA NPs on the activities of hepatic antioxidant enzymes in rats. (**A**): Catalase, (**B**): Glutathione peroxidase, (**C**): Glutathione reductase, and (**D**): Heme oxygenase. Data are expressed as Mean ± SD. * *p* < 0.05, significantly different when compared with a control group.

Iron is an essential trace element, and its systemic and cellular levels are tightly regulated. Following Fe-TA NPs administration in vehicle control rat groups, serum iron, TIBC, and transferrin saturation values were not determined to be different among experimental groups in comparisons with the control group. The administration of Fe-TA NPs at dosages of 0.55 and 5.5 mg/kg bw significantly increased iron content in the liver. However, no alteration in the ferritin levels of rat livers was found (Figure 3). In addition, there was no significant alteration of total, oxidized, and reduced glutathione contents in rat livers at 0.55 and 5.5 mg/kg bw dose of Fe-TA NPs. Notably, a high dose, 17.5 mg/kg bw, increased both the oxidized and reduced glutathione contents; however, this dosage did not alter their ratios when compared with the control group (Figure 4). This would indicate that an antioxidant response occurred at the above-mentioned dose in rat livers. The antioxidant enzyme activities in rat livers were further studied and results are presented in Figure 5. It was observed that 0.55 mg/kg bw doses of Fe-TA NPs decreased catalase activity, whereas doses of 17.5 mg/kg bw increased these levels in rat livers. Heme oxygenase activity was also increased by a dose of 17.5 mg/kg bw of Fe-TA NPs. Remarkably, GR and GPx activities were not found to be altered in rat livers (Figure 5).

## 4. Discussion

Theranostic nanoparticles allow for the simultaneous application of diagnostic and therapeutic functionalities in a single platform and aim to improve better clinical efficiency for patients. These particles can offer great promise in the diagnostic imaging, monitoring, and treatment of cancer. The integration of iron and TA as a self-assembled nanoparticle can facilitate iron in providing an MRI signal, as well as to inhibit tumor cell growth and enable TA to exert anticancer effects. Fe–TA NPs enhance an MRI contrast signal in hepatocellular carcinoma cell lines and liver preneoplasia in rats, as well as to exhibit an anti-proliferative effect on hepatocellular carcinoma cells via enhanced autophagic cell death [10,11]. The outcomes of the present study have indicated that the LD_50_ value for intraperitoneal administration of Fe–TA NPs in rats was more than 55 mg/kg bw. Furthermore, repeated various doses of Fe–TA NPs did not result in incidences of subacute toxicity, genotoxicity, and hepatocarcinogenicity in rats.

Toxicological effects of NPs are dependent upon certain physicochemical properties such as size, shape, and surface charge; as well as composition, stability, and potential interactions with other biological systems [27].The in vivo toxicity assessments considered the uptake, biodistribution, metabolism, and clearance of NPs. While in circulation, NPs can be coated by serum proteins and then engulfed by both circulating and tissue macrophages, mainly in the liver, spleen, lymph nodes, and bone marrow of rats. We found no systemic toxicity following Fe–TA NPs administration at a dose of 0.55–5.5 mg/kg bw in rats. Importantly, body and organ weight changes are an indicator of substance toxicity [28]. Fe–TA NPs in the present study were found to increase the relative weights of the ovaries and seminal vesicles by 5.5 and 1.1 mg/kg bw dose, respectively; however, they did not induce any histopathological changes in these organs. Previous studies have reported that NPs can exert a toxic effect on reproductive organs [29]. The growth and activity of these organs are regulated by sex hormones that can alter organ weight and histology [30,31]. Therefore, it could be assumed that Fe–TA NPs treatments may be associated with weak endocrine activity.

The hematopoietic system is one of the sensitive parameters for toxicity assessment. Bone marrow and lymphoid organs are major hemopoietic sites in adults, while the liver and spleen can take part in this process under certain conditions. NPs can accumulate and exert toxic effects in these organs leading to impaired hematopoiesis [32]. A previous study involving intraperitoneal injections of Fe_3_O_4_ NPs particles at a dose of 500 mg/kg 3 times a week for 5 weeks in rats resulted in erythropoiesis stimulation, neutrophil leukocytosis, and increased numbers of Kupffer cells [33]. However, Fe–TA NPs treated rats of both sexes revealed no abnormal alterations in extramedullary hematopoietic sites and hematology parameters.

Iron is transported in the plasma as transferrin and approximately 30% of transferrin are saturated with iron under normal conditions. Cells take up iron by transferrin receptor (TfR)-1 on the plasma membrane. Depending upon cellular iron requirements, excess iron can be stored as ferritin and can hold about 4500 iron atoms per molecule. The expression of ferritin becomes increased when cellular iron concentrations rise [34]. NPs mainly accumulate in the liver particularly in Kupffer cells [35]. NPs generally stay within these cells and then are slowly released. It was found that an intraperitoneal repeated-dose administration ranging from 0.22–17.5 mg/kg bw of Fe–TA NPs did not alter the serum iron status parameters that were evaluated in the present study. Although elevated liver iron content was observed in 0.55 and 5.5 mg/kg bw of Fe–TA NPs treated rats, neither iron overload nor hepatotoxicity occurred as was indicated by normal serum ALT levels. However, at a dose of 17.5 mg/kg bw, liver iron content was not altered. This outcome might be related to the iron chelating activity of TA in Fe–TA NPs [36]. The hormesis of various biological endpoints has been reported to be associated with TA and iron levels, as well as the presence of nanoparticles [12,37,38]. It appears that Fe–TA NPs exhibited a hormetic dose response that provided iron at low doses and chelated iron at high doses in the liver.

Moreover, NPs are known to be able to damage the glomerular membranes causing nephrotoxicity [35]. Glomerular filtration and renal excretory function appeared not to be affected by Fe–TA NPs in this study. Different degrees of proteinuria in male rats in both the control and Fe–TA NPs treated groups could be due to excretion of sex-dependent proteins since it has been described that proteinuria was observed in male rats during sexual maturation at about 8 weeks of age [39,40]. In addition, a probable reason for increased serum potassium levels in male rats that were given a dose of 5.5 mg/kg bw of Fe–TA NPs may be associated with the interaction of NPs with ion channels such as K^+^ and Na^+^ channels, as well as Na^+^/K^+^-ATPase, thereby altering channel activities and ion homeostasis [41].

Carcinogenesis is a multistep process that includes initiation, promotion, and progression steps [42]. A chemical substance can introduce carcinogenesis by causing unrepairable DNA damage in normal cells resulting in mutated or initiated cells. Under certain circumstances, this could progress to malignant neoplasm. NPs can interact with macromolecules including DNA, proteins, and lipids. DNA alkylation and oxidative damage can result in either gene mutation or chromosomal abnormalities such as numerical alteration, aneuploidy, and chromosome fragmentation. An assessment of the genotoxic potential of Fe–TA NPs through rat liver micronucleus test has demonstrated that Fe–TA NPs are not clastogenic, indicating non-genotoxicity. Fe–TA NPs was further evaluated in terms of hepatocarcinogenicity through a medium-term carcinogenicity test, which is known to be a reliable method used to predict the carcinogenic potential of chemicals. Since the proliferation rate of an adult liver is low, a two-third partial hepatectomy was carried out to enhance proliferation. Moreover, the exposure of certain chemicals to the liver after a partial hepatectomy could increase the sensitivity of the assay in detecting carcinogenicity [26]. The present study found that Fe–TA NPs did not produce glutathione *S*-transferase placental form positive foci, a hepatic preneoplastic lesion in rats [26], while also indicating a lack of evidence for sustained cell proliferation and the ability to resist cell apoptosis in the liver. Hence, these outcomes indicate that Fe–TA NPs are not associated with hepatocarcinogenicity in rats.

Oxidative damage or modifications of macromolecules can lead to toxicity and carcinogenicity. NPs toxicity is mainly mediated through ROS generation [27]. TA has been reported to be associated with both antioxidant and pro-oxidant effects [43,44]. Moreover, TA can reduce ferric iron to ferrous iron which can then generate deleterious hydroxyl radicals through a Fenton reaction. Cellular antioxidants can respond to oxidative stress through enzymatic and non-enzymatic mechanisms [45]. Catalase and glutathione peroxidase catalyze the removal of hydrogen peroxide. They show different affinities to H_2_O_2_. Catalase has low affinity to H_2_O_2_, while glutathione peroxidase has high affinity to H_2_O_2_. Catalase could undergo irreversible or reversible inactivation depending upon the H_2_O_2_ concentration. However, glutathione peroxidase exhibited lesser sensitivity to H_2_O_2_ induced inactivation. Baud et al. reported that after being exposed to H_2_O_2_ (100 µM for 1 h) in oligodendrocytes, catalase activity was reduced but glutathione peroxidase activity was not changed [46]. In the present study, the decreased catalase activity at low dose of Fe-TA NPs would likely be due to its inactivation by H_2_O_2_ [21]. At higher doses, increased total glutathione content and heme oxygenase activity were observed, which would indicate alleviation of the oxidative environment while also indicating that catalase could have taken part in the antioxidant response. These results are in line with the findings of Canli et al. who reported alterations in antioxidant enzymes activities, including those of superoxide dismutase, CAT, GPx, GR, and glutathione *S*-transferase, in rat livers after oral administration of some metal-oxide NPs at doses of 0.5–50 mg/kg/day for 14 days. Importantly, this would not necessarily indicate a dose-response relationship and correlation among the enzymes at each dose [47].

## 5. Conclusions

According to a toxicological evaluation of Fe-TA NPs, the LD_50_ value was greater than 55 mg/kg bw. Repeated intraperitoneal administration of Fe-TA NPs at up to 17.5 mg/kg bw demonstrated no obvious signs of systemic toxicity, genotoxicity, or hepatocarcinogenicity. Consequently, Fe-TA NPs might be safe for use in diagnostic and therapeutic purposes. Since this study emphasized only the early phase of hepatocarcinogenicity, further studies are needed to explore the effects on other phases of hepatocarcinogenesis.

## Figures and Tables

**Table 1:** Effect of 28-day administration of Fe-TA NPs on general observation and organ weights.

Parameters	Fe-TA NPs Treatment (mg/kg bw)					
	0	0.22	1.1	5.5	Satellite	
					0	5.5
**Female**						
**Food intake** **(g/day)**	14.3 ± 2.0	15.9 ± 2.3	14.5 ± 1.3	15.1 ± 1.8	15.4 ± 1.8	16.4 ± 2.1
**Water intake** **(mL/day)**	18.9 ± 3.2	19.7 ± 5.5	20.8 ± 5.1	21.0 ± 3.7	22.5 ± 1.9	19.0 ± 2.4
**IBW (g)**	120 ± 6.12	120 ± 8.66	121 ± 5.48	121 ± 4.18	120 ± 7.07	121 ± 2.24
**FBW (g)**	187 ± 4.47	193 ± 11.0	188 ± 17.2	193 ± 9.08	214 ± 15.5	204 ± 9.46
**Liver (g)**	6.36 ± 0.54	6.88 ± 0.30	6.53 ± 0.43	6.81 ± 0.47	6.94 ± 0.81	6.99 ± 0.48
**Spleen (g)**	0.37 ± 0.04	0.40 ± 0.03	0.36 ± 0.06	0.45 ± 0.04	0.39 ± 0.07	0.38 ± 0.03
**Kidneys (g)**	1.50 ± 0.06	1.57 ± 0.12	1.52 ± 0.14	1.49 ± 0.09	1.57 ± 0.17	1.51 ± 0.10
**Lungs (g)**	0.97 ± 0.12	0.89 ± 0.10	0.88 ± 0.08	0.90 ± 0.02	0.92 ± 0.14	0.83 ± 0.05
**Heart (g)**	0.60 ± 0.02	0.59 ± 0.05	0.56 ± 0.07	0.58 ± 0.07	0.62 ± 0.05	0.61 ± 0.03
**Thymus (g)**	0.38 ± 0.08	0.46 ± 0.03	0.43 ± 0.08	0.43 ± 0.10	0.46 ± 0.04	0.31 ± 0.04
**Pancreas (g)**	0.62 ± 0.16	0.68 ± 0.25	0.72 ± 0.29	0.92 ± 0.21	0.57 ± 0.07	0.69 ± 0.08
**Brain (g)**	1.60 ± 0.08	1.66 ± 0.06	1.64 ± 0.04	1.65 ± 0.06	1.36 ± 0.13	1.18 ± 0.03 #
**Adrenal (mg)**	64.0 ± 18.3	55.5 ± 9.06	60.8 ± 11.7	58.3 ± 3.61	64.9 ± 4.78	71.1 ± 13.8
**Ovary (mg)**	69.5 ± 15.4	91.1 ± 11.2	89.0 ± 12.5	110 ± 7.81 *	103 ± 31.2	80.7 ± 15.3
**Male**						
**Food intake** **(g/day)**	23.9 ± 2.6	26.9 ± 6.5	25.4 ± 5.2	23.4 ± 1.6	23.5 ± 1.7	23.8 ± 2.9
**Water intake** **(ml/day)**	27.9 ± 8.9	32.6 ± 6.8	32.4 ± 8.2	33.3 ± 5.1	30.0 ± 4.25	29.7 ± 4.22
**IBW (g)**	180 ± 12.3	180 ± 9.35	180 ± 6.12	180 ± 0.00	180 ± 14.1	180 ± 9.35
**FBW (g)**	321 ± 30.9	338 ± 23.9	314 ± 25.1	316 ± 9.62	365 ± 32.4	363 ± 18.6
**Liver (g)**	11.0 ± 1.33	12.0 ± 1.17	11.7 ± 1.56	11.0 ± 0.83	11.1 ± 1.91	11.0 ± 0.57
**Spleen (g)**	0.63 ± 0.14	0.64 ± 0.06	0.63 ± 0.07	0.67 ± 0.05	0.65 ± 0.14	0.66 ± 0.04
**Kidneys (g)**	2.46 ± 0.21	2.54 ± 0.15	2.62 ± 0.28	2.34 ± 0.08	2.38 ± 0.21	2.47 ± 0.31
**Lungs (g)**	1.18 ± 0.12	1.29 ± 0.18	1.38 ± 0.32	1.21 ± 0.15	1.13 ± 0.09	1.29 ± 0.20
**Heart (g)**	0.91 ± 0.15	0.93 ± 0.13	0.89 ± 0.07	0.88 ± 0.05	0.91 ± 0.05	0.96 ± 0.09
**Thymus (g)**	0.67 ± 0.14	0.67 ± 0.23	0.62 ± 0.09	0.62 ± 0.14	0.55 ± 0.14	0.48 ± 0.10
**Pancreas (g)**	0.79 ± 0.14	0.94 ± 0.22	0.87 ± 0.19	0.76 ± 0.22	0.73 ± 0.24	0.97 ± 0.17
**Brain (g)**	1.92 ± 0.09	1.85 ± 0.08	1.90 ± 0.09	1.82 ± 0.06	1.86 ± 0.10	1.84 ± 0.08
**Adrenal (mg)**	58.90 ± 6.32	55.00 ± 7.36	63.94 ± 17.90	64.36 ± 12.54	60.60 ± 10.06	66.58 ± 12.35
**Testes (g)**	3.34 ± 0.26	3.20 ± 0.23	3.30 ± 0.17	3.10 ± 0.19	3.65 ± 0.11	3.64 ± 0.17
**Prostate (g)**	0.39 ± 0.12	0.38 ± 0.09	0.41 ± 0.04	0.31 ± 0.06	0.44 ± 0.09	0.39 ± 0.05
**Epididymis (g)**	0.79 ± 0.12	0.78 ± 0.18	0.66 ± 0.12	0.63 ± 0.11	1.05 ± 0.07	1.03 ± 0.09
**Seminal (g)**	0.72 ± 0.11	0.76 ± 0.09	0.95 ± 0.09 *	0.84 ± 0.08	0.89 ± 0.19	0.81 ± 0.25

Values are expressed as Mean ± SD. (IBW: Initial body weight, FBW: Final body weight). * *p* < 0.05, significantly different when compared with the control. # *p* < 0.05, significantly different when compared with the control satellite.

**Table 2:** Effect of Fe-TA NPs on hematological profile in rats exposed to repeated dose toxicity tests.

Parameters	Normal Range	Fe-TA NPs Treatment (mg/kg bw)					
		0	0.22	1.1	5.5	Satellite	
						0	5.5
**Female**							
**RBC (×10^6^ cell/µL)**	4.6–9.2	7.3 ± 0.3	6.9 ± 0.6	7.6 ± 0.5	7.3 ± 0.4	8.5 ± 0.1	8.7 ± 0.6
**Hemoglobin (g/dL)**	11–19.2	15.7 ± 0.4	14.4 ± 1.2	16.1 ± 0.8	15.2 ± 0.8	18.1 ± 0.6	18.8 ± 1.1
**Hematocrit (%)**	36–53	48 ± 0.6	44 ± 3.2	49 ± 2.3	45.9 ± 2.0	52.9 ± 1.3	52.9 ± 2.7
**MCV (fL)**	48–70	66 ± 2.4	64 ± 1.3	65 ± 2.7	63 ± 1.1	62 ± 2.0	61 ± 2.0
**MCH (pg)**	16.0–23.1	21.7 ± 0.5	21.0 ± 0.8	21.3 ± 0.9	20.9 ± 0.8	21.3 ± 0.8	21.6 ± 0.6
**MCHC (g/dL)**	28.2–34.1	33.1 ± 0.7	32.7 ± 0.6	33.0 ± 0.3	33.2 ± 0.7	34.2 ± 0.7	35.5 ± 0.2
**RDW (%)**	10.0–16.0	12.2 ± 0.3	12.3 ± 0.8	12.2 ± 0.4	12.7 ± 0.5	12.6 ± 1.0	13.0 ± 0.9
**Reticulocytes (%)**	1.7–4.9	4.9 ± 1.6	3.8 ± 1.4	3.9 ± 0.8	3.7 ± 1.4	3.0 ± 1.0	2.9 ± 0.9
**WBC (×10^3^ cell/mm^3^)**	2–17	4 ± 1	3 ± 1	5 ± 1	5 ± 0	5 ± 1	4 ± 1
**Neutrophil (%)**	10–30	12 ± 3.9	12 ± 3.4	15 ± 10.4	12 ± 10.3	10 ± 1.0	16 ± 2.8
**Lymphocyte (%)**	65–85	83 ± 4.3	83 ± 4.30	79 ± 7.9	84 ± 8.5	85 ± 2.1	77.5 ± 2.4
**Monocyte (%)**	0–5	4 ± 2.5	4.8 ± 2.5	6 ± 2.95	4 ± 2.1	5 ± 0.1	7 ± 1.5
**Eosinophil (%)**	0–6	1 ± 1.2	0.4 ± 0.89	0.2 ± 0.45	0.0 ± 0.0	0.3 ± 0.5	0.0 ± 0.0
**Basophil (%)**	0–1	0.0 ± 0.0	0.0 ± 0.0	0.0 ± 0.0	0.0 ± 0.0	0.0 ± 0.0	0.0 ± 0.0
**PLT (×10^3^ cell/mm^3^)**	500–1300	787 ± 67	480 ± 337	762 ± 77	67.7 ± 206	793 ± 38	625 ± 315
**Male**							
**RBC (×10^6^ cell/µL)**	4.6–9.2	5.8 ± 0.2	5.8 ± 0.3	6.0 ± 0.2	5.8 ± 0.2	6.7 ± 0.8	6.4 ± 0.9
**Hemoglobin (g/dL)**	11–19.2	16.7 ± 0.3	16.7 ± 0.3	16.7 ± 0.4	16.1 ± 0.5	15.1 ± 0.6	14.7 ± 0.5
**Hematocrit (%)**	36–53	49.1 ± 1.4	48.3 ± 1.6	49.3 ± 1.4	46.8 ± 1.1	45.4 ± 1.8	44.0 ± 1.6
**MCV (fL)**	48–70	84.0 ± 1.6	83.2 ± 2.6	82.4 ± 1.9	81.1 ± 1.8	68.1 ± 6.3	70.2 ± 8.4
**MCH (pg)**	16.0–23.1	28.6 ± 0.8	28.7 ± 1.0	28.0 ± 0.5	28.0 ± 0.5	22.6 ± 2.1	23.5 ± 2.8
**MCHC (g/dL)**	28.2–34.1	34.0 ± 0.5	34.5 ± 0.4	33.9 ± 0.5	34.5 ± 0.3	33.4 ± 0.3	33.4 ± 0.1
**RDW (%**	10.0–16.0	10.9 ± 0.3	11.1 ± 0.7	11.2 ± 0.5	11.3 ± 0.6	14.6 ± 3.6	12.8 ± 3.4
**Reticulocytes (%)**	1.7–4.9	3.2 ± 2.1	3.0 ± 1.2	5.0 ± 1.2	5.4 ± 2.0	2.1 ± 1.7	2.3 ± 1.0
**WBC (×10^3^ cell/mm^3^)**	2–17	6 ± 1	6 ± 2	7 ± 1	7 ± 2	5 ± 3	5 ± 2
**Neutrophil (%)**	10–30	7 ± 2.1	10 ± 0.5	14 ± 3.5 *	15 ± 5.2 *	17 ± 0.9	15 ± 6.0
**Lymphocyte (%)**	65–85	89 ± 2.2	87 ± 0.9	86 ± 3.5	85 ± 5.2	82 ± 1.1	83 ± 6.3
**Monocyte (%)**	0–5	5 ± 0.8	3 ± 1.1 *	0 ± 0.0 *	0 ± 0.0 *	1 ± 0.5	2 ± 0.5
**Eosinophil (%)**	0–6	0 ± 0	0 ± 0	0 ± 0	0 ± 0	0 ± 0	0 ± 0
**Basophil (%)**	0–1	0 ± 0	0 ± 0	0 ± 0	0 ± 0	0 ± 0	0 ± 0
**PLT (×10^3^ cell/mm^3^)**	500–1300	770 ± 177	802 ± 151	770 ± 103	915 ± 216	731 ± 65.2	590 ± 129

Values are expressed as Mean ± SD. * *p* < 0.05, significantly different when compared with the vehicle control. (RBC, red blood cell; MCH, mean corpuscular hemoglobin; MCHC, mean corpuscular hemoglobin concentration; MCV, mean corpuscular volume; RDW, red cell distribution width; PLT, platelet; WBC, white blood cell).

**Table 3:** Effect of Fe-TA NPs on biochemical profiles in rats exposed to repeated dose toxicity tests.

Parameters	Normal Range	Fe-TA NPs Treatment (mg/kg bw)					
		0	0.22	1.1	5.5	Satellite	
						0	5.5
**Female**							
**Glucose (mg/dL)**	85–132	129 ± 14	144 ± 25	142 ± 13	167 ± 20	61 ± 21	173 ± 61
**Cholesterol (mg/dL)**	40–130	63 ± 10	70 ± 9	65 ± 8	67 ± 12	69 ± 4	68 ± 10
**Triglyceride (mg/dL)**	80–190	55 ± 12	49 ± 30	39± 8	41 ± 13	80 ± 11	55 ± 20
**BUN (mg/dL)**	10–21	24 ± 2	22 ± 3	18 ± 3 *	19 ± 2 *	25 ± 5	22 ± 2
**Creatinine (mg/dL)**	0.5–1.0	0.3 ± 0.1	0.2 ± 0.0	0.2 ± 0.0	0.2 ± 0.0	0.3± 0.0	0.3 ± 0.1
**Total protein (g/dL)**	6.3–8.6	7.1 ± 0.2	6.8 ± 0.6	7.0 ± 0.2	7.1 ± 0.3	8.5 ± 0.3	9.0 ± 0.5
**Albumin (g/dL)**	3.3–4.9	4.6 ± 0.1	4.3 ± 0.3	4.4 ± 0.2	4.4 ± 0.1	4.7 ± 0.2	4.7 ± 0.2
**AST (U/L)**	39–92	37 ± 17	42 ± 19	34 ± 7	28 ± 5.9	37 ± 12	42 ± 14
**ALT (U/L)**	17–50	117 ± 42	102 ± 16	122 ± 31	95 ± 26	96 ± 16	103 ± 20
**ALP (U/L)**	39–216	49 ± 7	55 ± 10	51 ± 10	54 ± 17	46 ± 6	33 ± 2
**Total bil (mg/dL)**	0.05–0.17	0.14 ± 0.02	0.09 ± 0.02	0.12 ± 0.06	0.12 ± 0.05	0.23 ± 0.03	0.22 ± 0.01
**Direct bil (mg/dL)**	0.03–0.07	0.08 ± 0.02	0.08 ± 0.01	0.09 ± 0.03	0.01 ± 0.03	0.09 ± 0.02	0.08 ± 0.01
**Uric acid (mg/dL)**	1.4–3.7	4.2 ± 0.6	3.5 ± 1.1	3.9 ± 0.7	4.8 ± 1.0	5.0 ± 2.0	4.5 ± 1.8
**Sodium (mmol/L)**	140–150	143 ± 2	141 ± 1	143 ± 1	142 ± 1	143 ± 1	144 ± 1
**Potassium (mmol/L)**	4–5.9	5.3 ± 0.7	5.9 ± 0.5	5.5 ± 0.4	5.6 ± 0.5	4.9 ± 0.2	5.2 ± 0.3
**Chloride (mmol/L)**	96–107	104 ± 2	105 ± 1	105 ± 1	105 ± 1	102 ± 2	104 ± 1
**TCO_2_ (mmol/L)**	13–27.1	20.6 ± 1.5	22.6 ± 1.3	22.4 ± 1.6	22.9 ± 2.7	22.6 ± 2.5	19.5 ± 1.5
**Male**							
**Glucose (mg/dL)**	85–132	214 ± 30	227 ± 44	290 ± 39	211 ± 45	176 ± 38	164 ± 23
**Cholesterol (mg/dL)**	40–130	66 ± 9	73 ± 20	64 ± 15	61 ± 9	73 ± 6	72 ± 10
**Triglyceride (mg/dL)**	80–190	40 ± 18	75 ± 24	47 ± 26	38 ± 12	75 ± 45	48 ± 23
**BUN (mg/dL)**	10–21	19 ± 2	18 ± 2	19 ± 2	17 ± 3	19 ± 3	17 ± 1
**Creatinine (mg/dL)**	0.5–1.0	0.8 ± 0.1	0.8 ± 0.1	0.8 ± 0.1	0.7 ± 0.0	0.8 ± 0.1	0.7 ± 0.0 #
**Total protein (g/dL)**	6.3–8.6	6.7 ± 0.2	6.7 ± 0.2	6.9 ± 0.2	6.6 ± 0.1	6.8 ± 0.3	6.5 ± 0.1
**Albumin (g/dL)**	3.3–4.9	3.7 ± 0.1	3.6 ± 0.1	3.7 ± 0.1	3.6 ± 0.1	3.6 ± 0.1	3.5 ± 0.1
**AST (U/L)**	39–92	114 ± 14	108 ± 13	114 ± 22	100 ± 19	116 ± 32	95 ± 14
**ALT (U/L)**	17–50	30 ± 5	33 ± 4	40 ± 10	31 ± 6	31 ± 5	26 ± 3
**ALP (U/L)**	39–216	103 ± 19	93 ± 4	96 ± 10	87 ± 23	75 ± 18	66 ± 16
**Total bil (mg/dL)**	0.05–0.17	0.07 ± 0.01	0.07 ± 0.01	0.05 ± 0.02	0.06 ± 0.01	0.08 ± 0.01	0.06 ± 0.02
**Direct bil (mg/dL)**	0.03–0.07	0.03 ± 0.01	0.02 ± 0.01	0.02 ± 0.00	0.03 ± 0.01	0.04 ± 0.01	0.04 ± 0.01
**Uric acid (mg/dL)**	1.4–3.7	5.3 ± 1.9	5.1 ± 1.4	7.3 ± 1.7	5.7 ± 0.8	2.8 ± 0.7	1.9 ± 0.6
**Sodium (mmol/L)**	140–150	145 ± 2	143 ± 4	144 ± 3	144 ± 1	146 ± 1	144 ± 1 #
**Potassium (mmol/L)**	4–5.9	5.7 ± 0.5	6.1 ± 0.8	6.7 ± 0.7	7.1 ± 0.7 *	5.2 ± 0.7	4.9 ± 0.6
**Chloride (mmol/L)**	96–107	100 ± 1	100 ± 3	101± 3	101 ± 2	101 ± 1	101 ± 1
**TCO_2_ (mmol/L)**	13–27.1	25.8 ± 2.0	27.1 ± 1.6	25.6 ± 2.1	25.6 ± 1.6	25.0 ± 1.1	27.4 ± 1.6 #

Values are expressed as Mean ± SD. * *p* < 0.05, significantly different when compared with the control. # *p* < 0.05, significantly different when compared with the satellite control. (BUN: blood urea nitrogen, AST: aspartate aminotransferase, ALT: alanine aminotransferase, ALP: alkaline phosphatase, bil: bilirubin, TCO_2_: total carbon dioxide test).

**Table 4:** Effect of Fe-TA NPs on micronucleus formation in rat livers.

Fe-TA NPs Treatment (mg/kg bw)	Body Weight Gain (%)	Total Number of (Per 1000 Hepatocytes)			Mitotic Index
		MN	MNH	BH	
**5% Tween 80**	30.8 ± 4.6	0.29 ± 0.39	0.29 ± 0.39	16.43 ± 2.82	0.72 ± 0.12
**Fe-TA NPs 55 mg/kg bw (Single dose)**	29.1 ± 4.8	0.68 ± 0.72	0.68 ± 0.72	18.60 ± 1.95	0.93 ± 0.12
**Fe-TA NPs 5.5 mg/kg bw (Single dose)**	32.4 ± 6.9	1.00 ± 0.93	1.00 ± 0.93	13.80 ± 0.84	0.79 ± 0.10
**Fe-TA NPs 5.5 mg/kg bw (Repeated doses)**	29.4 ± 4.2	0.30 ± 0.45	0.30 ± 0.45	16.00 ± 1.58	0.78 ± 0.13

Values are expressed as Mean ± SD. MN: Micronucleus, MNH: Micronucleated hepatocyte, BH: Binucleated hepatocytes.

**Table 5:** Effect of iron (III)–tannic acid nanoparticles on general observation, serum ALT level, preneoplastic lesions formation, cellular apoptosis, and proliferation in rat livers.

Parameters	Fe-TA NPs Treatment (mg/kg bw)			
	0	0.55	5.5	17.5
Final Body Weight (g)	495 ± 33	463 ± 19	466 ± 30	478 ± 49
Absolute Organ weight (g)				
Liver	15.77 ± 1.96	14.00 ± 1.86	14.76 ± 2.67	17.27 ± 2.28
Spleen	0.88 ± 0.06	0.82 ± 0.13	0.77 ± 0.06	0.80 ± 0.17
Kidneys	3.40 ± 0.35	3.25 ± 0.23	3.39 ± 0.14	3.40 ± 0.74
Serum ALT levels (U/L)	41.7 ± 14.8	37.5 ± 8.6	32.4 ± 6.0	39.0 ± 14.5
Number of GST-P positive foci/Liver area (cm^2^)	0.00 ± 0.00	0.00 ± 0.00	0.00 ± 0.00	0.00 ± 0.00
Area of GST-P positive foci (mm^2^)/Liver area (cm^2^)	0.00 ± 0.00	0.00 ± 0.00	0.00 ± 0.00	0.00 ± 0.00
Number of apoptotic cells/Liver area (mm^2^)	5.27 ± 4.50	6.19 ± 1.48	6.17 ± 3.43	5.14 ± 1.01
Number of PCNA-positive cells/Liver area (mm^2^)	8.31 ± 2.09	7.66 ± 1.87	8.95 ± 2.53	7.15 ± 2.16

Values are expressed as mean ± SD. ALT, alanine aminotransferase.

## Data Availability

All data presented in this study are available upon request from the corresponding author.
